# Image Formation and Resolution in Spatially Variant Coherent Imaging Systems

**DOI:** 10.3390/s26123733

**Published:** 2026-06-11

**Authors:** Junchang Li, Chung-Hsuan Huang, Jinbin Gui, Chau-Jern Cheng, Han-Yen Tu

**Affiliations:** 1 Department of Physics, Kunming University of Science and Technology, Kunming 650500, China; jinbingui@163.com; 2 Department of Electrical Engineering, Chinese Culture University, Taipei 11114, Taiwan; hcx4@ulive.pccu.edu.tw; 3 Institute of Electro-Optical Engineering, National Taiwan Normal University, Taipei 11677, Taiwan; cjcheng@ntnu.edu.tw

**Keywords:** coherent imaging, digital holographic microscopy, spatial resolution, space-variant

## Abstract

Since the invention of lasers, coherent imaging has been widely employed in digital holographic microscopy. Improving the resolution of the image field remains a key challenge for achieving high-precision measurements. However, due to the high coherence of the laser, the resolution of the wavefront at the image plane depends not only on the radius of curvature of the illumination wavefront, but also on the observation position and direction. Existing theoretical approaches, which provide only approximate calculations of the amplitude distribution of the image field, are insufficient for practical applications. In this study, a theoretical framework for calculating the complex wavefield at the image plane is established, and analytical expressions describing the spectral distribution as functions of observation position and direction are derived. The proposed theory is experimentally validated using digital holographic microscopy. The results show good agreement between theory and experiment, demonstrating that the proposed approach accurately characterizes the spectral and resolution variations in the image field. These findings provide a solid theoretical foundation for the optimal design of digital holographic microscopy systems and illumination wavefields.

## 1. Introduction

Coherent imaging systems play an important role in high-precision optical metrology, with applications in wafer surface metrology [1,2], defect inspection [3,4], nanoscale lithography alignment [5,6], and biomedical cell imaging [7,8]. Among these techniques, digital holographic microscopy (DHM) [9,10,11,12,13] is one of the most representative methods for precision measurement. In 2024, Jan Christoph Thiele and co-workers applied dark-field digital holographic microscopy to measure the scattering fields of protein monomers with physical sizes of 20–60 nm [14], demonstrating its potential for micro- and nanoscale measurements.

In these sensing and metrology applications, accurate prediction and control of spatial resolution are essential for reliable measurement. An inaccurate resolution model may cause misinterpretation of recorded signals, systematic dimensional errors, and non-optimal system design. Therefore, a quantitative framework that directly links illumination conditions, especially the wavefront curvature of the illumination beam, to the achievable resolution at different positions in the image plane would be useful for the design of coherent optical sensors and holographic measurement systems. Considerable effort has been devoted to improving the lateral and axial resolution of coherent imaging for three-dimensional objects. In this context, the resolvability of two points in object space after imaging into the image domain remains a fundamental criterion for evaluating the performance of resolution-enhancement methods [15,16].

The space-variant nature of optical image formation has been investigated in earlier studies, such as the work of Lohmann and co-workers on space-variant image formation [17] and related studies by Welford and others [18]. Sitter and Rhodes proposed a space-invariant model for certain space-variant three-dimensional imaging systems, showing that some space-variant image-formation problems can be simplified through an appropriate coordinate transformation and consideration of the imaging geometry [19]. These studies indicated that practical optical imaging systems cannot always be fully described by a simple space-invariant model. More recently, similar issues have also been discussed in digital holography, including investigations of space-variance effects in holographic imaging systems [20] and two-point resolution in defocused coherent imaging systems [21]. However, a quantitative description of how the image-plane spectral distribution varies with observation position and direction remains insufficient. Therefore, the present work further derives explicit spectral-distribution formulas for coherent imaging systems and validates them experimentally using digital holographic microscopy.

Consistent with these previous studies, investigations in DHM have shown that the resolution of the imaging field depends not only on the observation position but also on the illumination direction of the incident wave [22,23]. In practice, imaging targets have finite physical dimensions. When image resolution is evaluated using two object points near the optical axis, the high coherence of laser illumination causes surrounding object points to influence the image wavefront of the two points under investigation [24]. Therefore, evaluating imaging field resolution solely based on the images of two near-axis object points is theoretically insufficient.

Since amplitude and phase have equal physical significance in optical metrology, in 2016, Horstmeyer’s group proposed in *Nature Photonics* the use of amplitude and phase Siemens star targets to experimentally observe the imaging field distribution in different regions of the image plane [25]. They also suggested developing mathematical methods to extract complex wavefront information from digitally reconstructed holograms. Nevertheless, most existing studies still focus on reporting experimental resolution improvements between two points in specific imaging regions [16,26,27]. Thus, further refinement of the theoretical model and the establishment of an appropriate evaluation methodology remain important issues.

In coherent imaging, this challenge is further complicated by the lack of a rigorous theoretical framework for accurately calculating the complex wavefront of the imaging field. For coherent imaging systems composed of multiple optical elements, researchers generally rely on angular spectrum diffraction and related propagation methods to trace the complex wavefront between optical elements [16], which makes the theoretical analysis highly complicated. In this study, the object under inspection is regarded as being composed of numerous object points, and the image field is considered as the coherent superposition of the images formed by these object points. From this viewpoint, developing a calculation theory for the complex wavefront of the imaging field and analyzing resolution from the perspective of the image field spectrum can provide results that more closely reflect practical imaging conditions. Accordingly, two classical coherent imaging theories are examined in this work.

The first imaging formulation is based on a classical coherent imaging framework reported by Born and Wolf [28]. For relatively small objects, this framework assumes the existence of an isoplanatic region in which a coherent transfer function mainly determined by the system aperture function can be defined. Within this region, the complex wavefront of the imaging field can be described using a convolution-based expression. Accordingly, the coherent imaging system can be treated approximately as linear and space-invariant. Nevertheless, when extending this framework to practical imaging systems, the relationship between the aperture function and the underlying physical system parameters may require further clarification.

The second formulation is based on the Fourier optics framework presented by Goodman [29]. In this treatment, the amplitude transfer function is defined by the pupil function, and the amplitude at the image plane is derived through an approximate treatment of the system impulse response. Image formation is thus expressed as the convolution of the ideal image with the system impulse response, such that the system is again modeled as linear and space-invariant. Since several approximations are introduced in the derivation, this formulation is generally more suitable for relatively small objects.

Because of the complexity of coherent imaging calculations, the approximate coherent imaging formula reported in ref. [29] is still widely used as a theoretical tool. To further investigate the resolution of the imaging field, we combine optical matrix analysis with diffraction theory and derive a formulation for calculating the complex wavefront of the imaging field without approximating the system impulse response [30]. The feasibility of this formulation has been verified through a series of experimental studies [30,31,32,33]. Mathematical analysis of this formulation shows that coherent imaging systems are not linear and space-invariant. This finding provides a more reasonable explanation for the spatial variation in resolution observed across the image plane. Recent studies further indicate that, for a given imaging system, the optical spectrum in the image plane exhibits a relatively complex functional dependence. It is determined not only by the illumination wavelength, the size of the exit pupil, and the distance between the exit pupil and the image plane, but also by the observation position and the illumination direction of the incident wave.

To validate the theoretical results, experimental verification was performed using DHM. From the perspective of imaging system design, the results of this study may provide useful guidance for the analysis and optimization of coherent imaging systems. For a given microscope objective, relay lens configuration, and image sensor array, the optical parameters of the system can be used to identify a suitable illumination wavefront for reducing spatial variations in resolution across the sensor field, thereby improving measurement consistency for targets located at different positions. For applications requiring enhanced lateral resolution in localized regions, the analysis also suggests that adjusting the target position and the illumination wavefront curvature may improve local imaging performance without modifying the hardware configuration. In addition, the proposed formulation can serve as a forward model for computational sensing, supporting the development of spatially adaptive reconstruction methods that take position- and direction-dependent point spread functions into account. Recent computational imaging studies have shown that space variance in optical systems can be partially compensated by advanced reconstruction algorithms, such as iterative phase retrieval methods incorporating space-variant point spread functions [34]. In this context, the proposed spectral-distribution analysis provides a physically interpretable way to relate the space-variant imaging behavior to the optical system parameters, observation position, and illumination direction, and may therefore help simplify the modeling and optimization of coherent and holographic imaging systems.

The remainder of this paper is organized as follows. Section 2 presents the theoretical results on the spectral distribution of the image field in coherent imaging systems. Section 3 provides the experimental validation. Section 4 presents a discussion of the research results. To facilitate practical applications, a computational formula applicable to the complex wavefront of the image field for large sampling arrays is derived. Section 5 concludes the paper. Appendix A gives the theoretical derivation of the imaging formula for coherent imaging systems.

## 2. Spectrum Distribution of Coherent Imaging System

In the Cartesian coordinate system *O-xyz*, with the *z*-axis taken as the optical axis, the optical field at the object plane can be written as $U_{o}(x,y)$ when the object plane is illuminated by a unit-amplitude plane wave propagating along the *z*-axis. A widely used coherent imaging formula is [29]:

(1) $$U\left(x,y\right)=F^{-1}\left\{F\left\{-\frac{1}{A}U_{o}\left(\frac{x}{A},\frac{y}{A}\right)\right\}P\left(-\lambda d_{pi}f_{x},-\lambda d_{pi}f_{y}\right)\right\}$$

where U(x,y) is the amplitude of the optical field in the image plane; F and $F^{-1}$ denote the two-dimensional Fourier transform and inverse Fourier transform, respectively; A is the lateral magnification; $-\frac{1}{A}U_{0}\left(\frac{x}{A},\frac{y}{A}\right)$ represents the ideal image; λ is the wavelength; $d_{pi}$ is the distance from the exit pupil to the image plane; $\left(f_{x},f_{y}\right)$ are the spatial-frequency coordinates; and $P\left(-\lambda d_{pi}f_{x},-\lambda d_{pi}f_{y}\right)$ is the amplitude transfer function defined by the exit pupil P(x,y) of the optical system. Equation (1) implies that the coherent imaging system is linear and space-invariant. Because the exit pupil P(x,y) is usually circularly symmetric, the amplitude transfer function physically acts as a low-pass filter on the spectrum of the ideal image. If the exit-pupil diameter is $D_{pi}$, the highest spatial frequency that can be transmitted by the amplitude transfer function is given by [29]:

(2) $$f_{max}=\left|\frac{D_{pi}}{2\lambda d_{pi}}\right|$$

According to Equation (2), for a given circularly symmetric imaging system, the resolution of the image field is predicted to be a constant independent of the observation position and observation direction.

Since a practical circularly symmetric imaging system can usually be described by the optical matrix $\left[\begin{array}{cc} A & 0\\ C & 1/A \end{array}\right]$ [29], matrix optics can be combined with diffraction theory to derive a more general expression for the complex wavefront of the image field. Letting k=2π/λ, where λ is the wavelength, and assuming unit-amplitude plane wave illumination of the object plane, the resulting expression is given by (see Appendix A for the derivation process):

(3) $$U\left(x,y\right)=exp\left[\frac{jk}{2d_{pi}}\left(x^{2}+y^{2}\right)\right]\times F^{-1}\left\{F\left\{-\frac{1}{A}U_{o}\left(\frac{x}{A},\frac{y}{A}\right)exp\left[j\frac{k}{2}\left(\frac{C}{A}-\frac{1}{d_{pi}}\right)\left(x^{2}+y^{2}\right)\right]\right\}\times P\left(-\lambda d_{pi}f_{x},-\lambda d_{pi}f_{y}\right)\right\}$$

where j represents $\sqrt{-1}$. Let $d={\left(\frac{C}{A}-\frac{1}{d_{pi}}\right)}^{-1}$; then, expanding the Fourier transform in Equation (3) yields:

(4) $$F\left\{-\frac{1}{A}U_{o}\left(\frac{x}{A},\frac{y}{A}\right)exp\left[\frac{jk}{2d}\left(x^{2}+y^{2}\right)\right]\right\}=\iint_{-\infty }^{\infty }\left\{-\frac{1}{A}U_{o}\left(\frac{x}{A},\frac{y}{A}\right)exp\left[\frac{jk}{2d}\left(x^{2}+y^{2}\right)\right]\right\}exp\left[-j2\pi \left(xf_{x}+yf_{y}\right)\right]dxdy$$

In Equation (4), if we define $f_{x}=\frac{x_{d}}{\lambda d}$, $f_{y}=\frac{y_{d}}{\lambda d}$, and compare it with the Fresnel diffraction integral of an ideal image propagating over a distance d:

(5) $$U\left(x_{d},y_{d}\right)=\frac{exp\left(jkd\right)}{j\lambda d}exp\left[\frac{jk}{2d}\left(x_{d}^{2}+y_{d}^{2}\right)\right]\times \iint_{-\infty }^{\infty }\left\{-\frac{1}{A}U_{o}\left(\frac{x}{A},\frac{y}{A}\right)exp\left[\frac{jk}{2d}\left(x^{2}+y^{2}\right)\right]\right\}exp\left[-j2\pi \left(x\frac{x_{d}}{\lambda d}+y\frac{y_{d}}{\lambda d}\right)\right]dxdy$$

**Figure 1:**
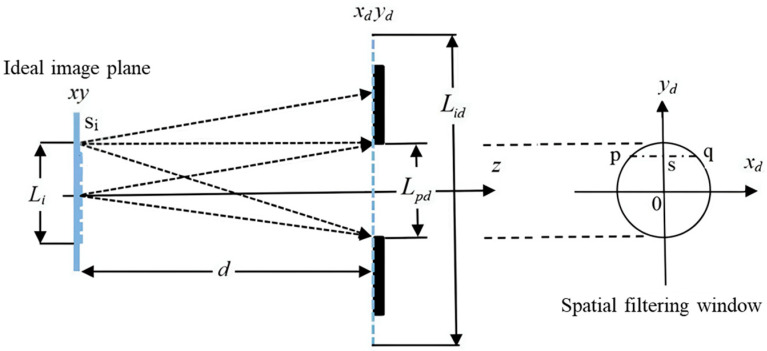
Schematic illustration of the influence of the coherent transfer function on the spectrum of the image field.

It can be readily seen that the magnitude of Equation (4) is λd times that of Equation (5). Therefore, the spectral amplitude distribution obtained from the Fourier transform of Equation (4) is similar to the amplitude distribution of the Fresnel diffraction field produced by propagation of the ideal image over a distance d. Accordingly, the coherent transfer function P in Equation (3) can be interpreted physically as being analogous to a “spatial filter” formed by the diffraction of the ideal image over a distance d [33]. Based on this physical interpretation of the coherent transfer function, Figure 1 schematically illustrates its effect on the spectrum of the image field. Let the *z*-axis of the coordinate system *O-xyz* be the optical axis of the imaging system, and let z=0 denote the ideal image plane. Let $L_{pd}$ be the diameter of the spatial filtering window, whose front view is shown on the right-hand side of the figure. Here, s denotes the geometric projection point on the *y*-axis corresponding to a differential element $s_{i}$ located at ${y=y}_{i}$ on the *y*-axis of the ideal image plane, as obtained from the Fourier transform calculation in Equation (5). The horizontal dashed line passing through point s intersects the boundary of the filtering window at points p and q.

According to angular spectrum diffraction theory, in the *yz*-plane, the arrow from $s_{i}$ to the lower boundary of the filtering window corresponds to the illumination direction of the maximum spectrum component that can pass through the filtering window in the negative *y*-direction, whereas the arrow from $s_{i}$ to the upper boundary corresponds to the propagation direction of the minimum spectrum component that can pass through the filtering window in the positive *y*-direction. If this problem is considered in three-dimensional space, the two arrows from $s_{i}$ toward q and p correspond to the maximum spectrum components of the differential element in the positive and negative *x*-directions, respectively. This implies that, except at the origin of the image plane, the spectral content associated with each observation region varies both spatially and directionally. A quantitative analysis of this property is presented below using discrete Fourier transform (DFT) theory.

Let the computational plane in Equation (4) be a square sampling grid of size N×N with a width of $L_{i}=2y_{i}$. The corresponding width of the frequency plane is $D_{f}=N/L_{i}$. When Equation (5) is used, the observation plane $\left(x_{d},y_{d}\right)$ after propagation over a distance d has a width of $L_{id}=\left|\frac{\lambda dN}{L_{i}}\right|$ [30]. If $D_{pi}$ denotes the diameter of the exit pupil, then the exit pupil forms a filtering window in the spectrum with a diameter of $D_{p}=\left|\frac{D_{pi}}{\lambda d_{pi}}\right|$, corresponding to a spatial filtering window with a diameter of $L_{pd}=\frac{D_{p}}{D_{f}}L_{id}=\left|\frac{D_{pi}d}{d_{pi}}\right|$. According to the geometric relationship shown in Figure 1, the highest spatial frequency that can be transmitted by the transfer function in the positive and negative *y*-directions is given by

(6) $$f_{max}^{\prime }\left(y_{i}\right)=\frac{L_{pd}\pm L_{i}}{2\lambda \left|d\right|}=\frac{D_{pi}}{2\lambda \left|d_{pi}\right|}\pm \frac{y_{i}}{\lambda \left|d\right|}$$

In these expressions, the positive sign corresponds to the negative *y*-direction, whereas the negative sign corresponds to the positive *y*-direction. Since the coordinate directions of the ideal image plane and the filtering plane can be chosen arbitrarily, $y_{i}$ in the above expressions may be regarded as the distance $r_{i}$ from point $s_{i}$ to the center of the image plane. Under this interpretation, the “+” sign is taken for the spectral component directed toward the optical axis, whereas the “−” sign is taken for the spectral component directed away from the optical axis.

**Figure 2:**
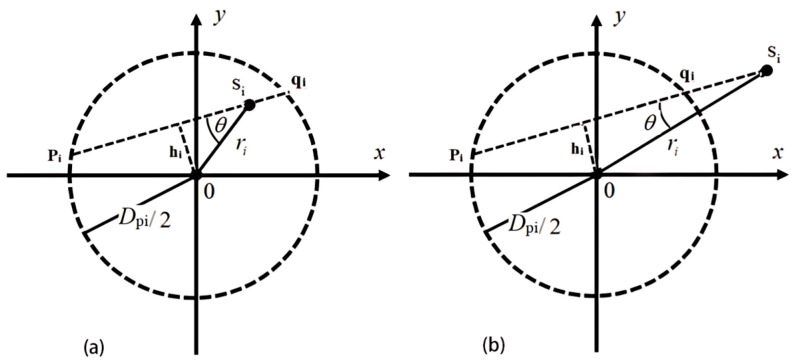
Illustrates the generalized spectral analysis of a point $s_{i}$ (**a**) inside and (**b**) outside the projected exit pupil.

An analysis of Equation (6) shows that $D_{pi}$ is the diameter of the exit pupil. Accordingly, for observation along the optical axis direction, the spectral value represented by the first term on the right-hand side is proportional to the pupil diameter. Following the same approach used in Figure 1, but replacing the diameter of exit pupil with the length of a chord of the exit pupil, Equation (6) can be extended to describe the maximum spatial frequency available in the observation region along the chord direction. Figure 2 illustrates this generalized case. In the figure, x and y denote the coordinates on the image plane, and the dashed circle represents the projection of the exit pupil onto the image plane. For an arbitrary point $s_{i}$ on the image plane, the corresponding chord is defined as the line passing through points $p_{i}$ and $q_{i}$.

Figure 2a,b show the spectral analysis diagrams for the cases where point $s_{i}$ is located inside and outside the projected region of the exit pupil, respectively. Let $h_{i}=r_{i}\sin\theta$ denote the distance from the origin of the image plane to the line connecting $p_{i}\to q_{i}$. Since the length of the line $p_{i}\to q_{i}$ is $\sqrt{{\left(D_{pi}/2\right)}^{2}-{r_{i}}^{2}{sin}^{2}\theta }$, and the angle θ on the side opposite to the line $s_{i}\to p_{i}$ is taken to be negative, a derivation similar to that in Figure 1 shows that when point $s_{i}$ is located inside the projected region of the exit pupil, the maximum transmitted spatial frequency is given by

(7) $$f_{max1}\left(r_{i},\theta \right)=\frac{\sqrt{{\left(D_{pi}/2\right)}^{2}-{r_{i}}^{2}{sin}^{2}\theta }}{\lambda \left|d_{pi}\right|}\pm \frac{r_{i}\cos\theta }{\lambda \left|d\right|}$$

The “+” sign corresponds to observation from $s_{i}$ toward $p_{i}$, while the “−” sign corresponds to the observation direction pointing to $q_{i}$.

When point $s_{i}$ lies outside the projected region of the exit pupil, only when $\left|\theta \right|<\sin^{-1}\left(\sqrt{\frac{D_{pi}}{2r_{i}}}\right)$ is satisfied can the angular-spectrum direction line emitted from the ideal-image observation point $s_{i}$ enter the spatial filtering window. In this case, the distances from $s_{i}$ to points $p_{i}$ and $q_{i}$ are $r_{i}\cos\theta +\sqrt{{\left(D_{pi}/2\right)}^{2}-{r_{i}}^{2}{sin}^{2}\theta }$ and $r_{i}\cos\theta -\sqrt{{\left(D_{pi}/2\right)}^{2}-{r_{i}}^{2}{sin}^{2}\theta }$, respectively. The spectral component along the direction from $s_{i}$ to $p_{i}$ is then described by Equation (8), in which the symbol “±” determines the maximum and minimum values.

(8) $$f_{\pm max2}\left(r_{i},\theta \right)=\left\{\begin{array}{c} \frac{r_{i}\cos\theta \pm \sqrt{{\left(D_{pi}/2\right)}^{2}-{r_{i}}^{2}{sin}^{2}\theta }}{\lambda \left|d_{pi}\right|}\;\;\;\;\;\;\;\;\;\;\;\;\;\;\left|\theta \right|<\sin^{-1}\left(\sqrt{\frac{D_{pi}}{2r_{i}}}\right)\\ 0\;\;\;\;\;\;\;\;\;\;\;\;\;\;\;\;\;\;\;\;\;\;\;\;\;\;\;\;\;\;\;\;\;\;\;\;\;\;\;\;\;\;\;\;\;\;\;\;\;\;\;\;\;\;\;\;\;\;\;\;\;\;\;\;\;\;\left|\theta \right|\geq \sin^{-1}\left(\sqrt{\frac{D_{pi}}{2r_{i}}}\right) \end{array}\right.$$

It is readily seen that, when the distance between the observation point in the image plane and the optical axis approaches zero, i.e., $r_{i}\to 0$, the nonzero terms in Equations (7) and (8) can be approximated by $\left|\frac{D_{pi}}{2\lambda d_{pi}}\right|$. In this sense, Equation (2) becomes the limiting case of Equations (7) and (8) as $r_{i}\to 0$. The following analysis shows that, for Equation (7), the value of $\left|d\right|$ has an important influence on the variation in the spectrum with the observation position in the image plane.

The above analysis has been carried out under plane wave illumination. To extend the discussion to a more general case, we further assume that the object plane is illuminated by a unit-amplitude spherical wave with a wavefront radius of $R_{e}$. This spherical wave illumination is equivalent to plane wave illumination with a thin lens of focal length $f=-R_{e}$ placed at the object plane. Under this equivalence, the optical matrix of the imaging system can be written as

(9) $$\left[\begin{array}{cc} A^{\prime } & 0\\ C^{\prime } & 1/A^{\prime } \end{array}\right]=\left[\begin{array}{cc} A & 0\\ C & 1/A \end{array}\right]\left[\begin{array}{cc} 1 & 0\\ -1/f & 1 \end{array}\right]=\left[\begin{array}{cc} A & 0\\ C-1/\left(Af\right) & 1/A \end{array}\right]$$

The above result shows that the magnification A of the imaging system remains unchanged, whereas the matrix element $C^{\prime }$ becomes C−1/(Af). Accordingly, in Equation (3), the transfer function can be interpreted as a “spatial filter” associated with Fresnel diffraction over a propagation distance of $d={\left(\frac{C^{\prime }}{A}-\frac{1}{d_{pi}}\right)}^{-1}$. Therefore, Equations (7) and (8) can be extended to the case of spherical wave illumination.

An examination of Equations (7) and (8) shows that, when $|d|\gg r_{i}$ and $D_{pi}\gg r_{i}$, the variation in the image field spectrum with observation position and observation direction becomes small, and the resolution in the image plane can be approximated as a constant. Based on this analysis, for a given optical system, a quantitative investigation can then be carried out to determine an optimal illumination wavefront radius $R_{e}$ such that a more uniform resolution distribution can be obtained in the image plane.

When $\left|d\right|$ is sufficiently large, the following condition must hold:

(10) $$\left|\frac{C^{\prime }}{A}-\frac{1}{d_{pi}}\right|=\left|\frac{C-1/\left(Af\right)}{A}-\frac{1}{d_{pi}}\right|\approx 0$$

Solving this condition yields

(11) $$f\approx \frac{d_{pi}}{Cd_{pi}A-A^{2}}=-R_{e}$$

When the illumination wavefront radius $R_{e}$ approximately satisfies the above condition, the Fourier transform result in Equation (3) approaches the spectrum of the ideal image, and the transfer function approximately behaves as a low-pass filter. Under such conditions, the detector plane can capture the complete image, and the image plane can also exhibit better spectral uniformity.

## 3. Theoretical Simulation and Experimental Validation

Since the high resolution of the image field is closely related to the highest spatial frequency component available in the observation region, experiments were performed to further verify the above theory. A 40-sector Siemens star (Ready Optics, Los Angeles, CA, USA; model: 2017a Star) was used as the test sample for DHM reconstruction under plane wave illumination and spherical wave illumination, approximately satisfying Equation (11). According to the manufacturer’s specifications, the Siemens star is fabricated as a high-contrast chrome pattern on a quartz glass substrate. The outside target diameter is 2 mm, and the pattern contains 40 spoke-space pairs. The specified minimum resolution is 300 nm per line pair, corresponding to a 150 nm single spoke, with circular breaks covering spatial periods from 128 μm to 0.3 μm per pair.

In the experimental procedure, the center of the Siemens star was first aligned with the optical axis, and a digital hologram was recorded. The Siemens star was then laterally translated in the object plane, and an additional hologram was recorded after its center had been shifted away from the optical axis. After identifying the center position of the Siemens star in the reconstructed image, the image field was calculated theoretically according to Equations (1) and (3). By comparing the experimental measurements with the theoretical simulations, the variation in resolution of the image field across different regions of the image plane was examined.

### 3.1. Experimental Setup

**Figure 3:**
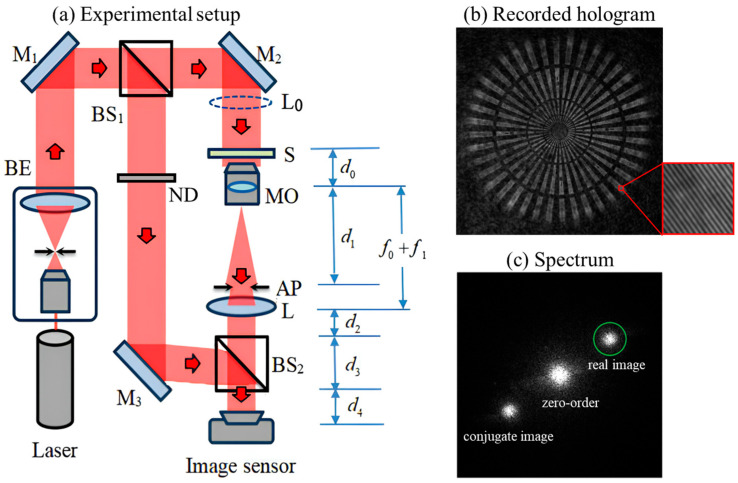
(**a**) Schematic of the microscopic digital holographic system for theoretical analysis and experimental validation. The blue dashed ellipse denotes lens $L_{0}$, which position was adjusted along the optical axis to realize a spherical wave illumination. (**b**) Recorded off-axis hologram, where the red box shows an enlarged view of the interference fringes, and (**c**) its corresponding spectrum.

Figure 3 shows the optical configuration of the DHM system. A He-Ne laser with a wavelength of λ= 632.8 nm (Uniphase, St. Charles, IL, USA; model: 1145P; 21 mW) is used as the coherent illumination light source. The laser is operated in continuous wave mode. A laser beam is converted into a plane wave by the beam expander (BE) and is then reflected by the mirror M_1_ toward the beam splitter (BS_1_). According to the manufacturer’s specifications, both BS_1_ and BS_2_ used in the experimental setup have a nominal transmitted-to-reflected intensity ratio of 50:50. The beam transmitted horizontally through BS_1_ is reflected by the mirror M_2_ and illuminates the object plane (S). The object wave is collected by a microscope objective (MO) with a focal length of $f_{0}=$ 16 mm and a numerical aperture (NA) of 0.25, and is then directed to the aperture (AP). After passing through the aperture, the object wave propagates toward lens (*L*), which has a diameter of $D_{1}=$ 50.8 mm and a focal length of $f_{1}=$ 180 mm. The wave emerging from the lens then passes through beam splitter (BS_2_), which has a refractive index of *n* = 1.5151 and a thickness of $d_{3}=$ 25.4 mm, and finally forms an image on the image sensor (IS). The image sensor (Pixoel, Taipei City, Taiwan; model: U3-34L0XCP-M-GL, Monochrome) is composed of a 3552 × 3552 pixel array with a pixel size of 2 μm, 8-bit digitization, and a frame rate of approximately 17 fps. Meanwhile, the beam reflected downward by BS_1_ is successively reflected by mirror M_3_ and BS_2_, forming a reference wave that is slightly tilted with respect to the optical axis before reaching the image sensor. Since the object beam passes through more optical elements and the sample, its intensity may be lower than that of the reference beam at the image sensor. Therefore, a neutral density filter was placed in the reference beam path to adjust the reference beam intensity. This adjustment made the object and reference beam intensities before the image sensor approximately balanced, providing more favorable interference conditions for hologram recording. The reference wave interferes with the object image at the image sensor, and the resulting off-axis digital hologram is recorded by the image sensor, as shown in Figure 3b. The corresponding spectrum is shown in Figure 3c. The tilt angles of the reference beam in the *x*- and *y*-directions were approximately 4.5° and −3.2°, respectively. The exposure time used for recording the holograms was 15 ms. The lens $L_{0}$ with a focal length of 100 mm, indicated by dashed lines in the figure, is inserted only in the spherical wave illumination experiment. Before inserting the Siemens star target, an optical flat was used to assist the alignment of the illumination wavefront. The spacing of the interference fringes reflected from the optical flat was adjusted to be as large as possible, indicating that the illumination wavefront at the object plane was sufficiently close to a plane wave. In addition, because the exact sensor detection plane is difficult to determine directly, the object-plane position used in the theoretical calculation was determined using measurable system parameters together with matrix optics. The system magnification was also cross-checked by measuring the radii of the opaque concentric rings in the reconstructed Siemens star image.

In the experimental system, considering the lens mount, the actual diameter of lens *L* is 50.1 mm. The insertion of the circular aperture AP, with a transmissive opening diameter of 4.6 mm, serves two purposes. First, it is used to verify that Equation (3) is not restricted by the size of the exit pupil of the optical system and can still accurately calculate the optical wavefield in the image plane. Second, by optimizing the spherical wave illumination on the object plane, the complete image can be detected in the image plane while the variation in resolution is relatively reduced.

For the experimental system, let $d_{s}=d_{2}+d_{3}/n+d_{4}$. The optical transfer matrix from the object plane to the image sensor can then be written as Equation (12).

(12) $$\left[\begin{array}{cc} A & 0\\ C & 1/A \end{array}\right]=\left[\begin{array}{cc} 1 & d_{s}\\ 0 & 1 \end{array}\right]\left[\begin{array}{cc} 1 & 0\\ -1/f_{1} & 1 \end{array}\right]\left[\begin{array}{cc} 1 & f_{0}+f_{1}\\ 0 & 1 \end{array}\right]\left[\begin{array}{cc} 1 & 0\\ -1/f_{0} & 1 \end{array}\right]\left[\begin{array}{cc} 1 & d_{0}\\ 0 & 1 \end{array}\right]\;\;\;\;\;\;\;\;\;\;\;\;\;\;\;\;\;\;\;\;\;\;\;\;\;\;\;\;\;\;\;\;\;\;\;\;\;\;\;=\left[\begin{array}{cc} -f_{1}/f_{0} & d_{0}\left(1-d_{s}/f_{1}\right)+\left(1-d_{0}/f_{0}\right)\left[d_{s}+\left(f_{0}+f_{1}\right)\left(1-d_{s}/f_{1}\right)\right]\\ 0 & -f_{0}/f_{1} \end{array}\right]$$

Equation (12) shows that, regardless of the values of $d_{0}$ and $d_{s}$, the matrix element C is always zero. Therefore, the image field is always an inverted image with a transverse magnification of $A=-f_{1}/f_{0}$. Furthermore, for a properly aligned 4-f imaging system, once either $d_{0}$ and $d_{s}$ is specified, the other can be determined from

(13) $$d_{0}\left(1-d_{s}/f_{1}\right)+\left(1-d_{0}/f_{0}\right)\left[d_{s}+\left(f_{0}+f_{1}\right)\left(1-d_{s}/f_{1}\right)\right]=0$$

In the experiment, careful alignment was carried out before inserting the Siemens star in order to ensure that the MO and *L* formed a well-aligned 4-f imaging system. The beam passing through BE and the wave transmitted through *L* were both adjusted to be collimated and to propagate along the optical axis. After the Siemens star was inserted, a digital hologram was recorded by the image sensor. To minimize the measurement error associated with the positions of the optical elements, we directly measured the distance from lens *L* to the image sensor IS, giving $d_{2}+d_{3}+d_{4}=189\;mm$. Based on this measurement, $d_{s}=d_{2}+d_{3}/n+d_{4}=180.36\;mm$ was obtained and was substituted into Equation (13), yielding $d_{0}\approx 16\;mm$.

Because the resolution in coherent imaging system depends on the phase relationship between the two image points under consideration, the Rayleigh criterion originally developed for incoherent imaging is no longer strictly applicable [15,29]. Nevertheless, following the discussion in ref. [15], it can still be used as a useful reference for examining the resolution characteristics of the experimentally reconstructed images.

According to the Rayleigh criterion [29], when the exit pupil has a diameter of $D_{pi}$, the minimum resolvable distance is $d_{min}=1.22\lambda d_{pi}/D_{pi}$. For the 40-sector Siemens star image used in the experiment, the minimum resolvable radius $r_{\min}$ satisfies $d_{\min}=2\pi r_{\min}/80$. With the aperture stop inserted, geometrical imaging analysis of the aperture stop by *L* shows that the exit pupil of the optical system is located 43.49 mm in front of *L*, with a diameter of $D_{pi}=$ 5.59 mm. The distance from the exit pupil to the image plane is therefore $d_{pi}=d_{s}+43.448\;\mathrm{mm}=223.81\;\mathrm{mm}$. Under these conditions, the minimum resolvable distance and radius are $d_{\min}=$ 0.03 mm and $r_{\min}=$ 0.38 mm, respectively. To better observe the variation in resolution with observation position, the amplitude variation along a circular ring of radius $R_{t}=0.64\;r_{\min}\approx 0.24\;\mathrm{mm}$ in the image plane was analyzed. To enable effective extraction of the image field spectrum, the tilt angle of the reference beam was adjusted such that the spectrum of the real and conjugate images was located near the centers of the first and third quadrants of the spectrum, respectively. This configuration facilitates spectral filtering and reduces interference from the zero-order component. Furthermore, during numerical reconstruction, the local average of the hologram intensity was estimated and subtracted to suppress the zero-order diffraction contribution, resulting in improved reconstructed image quality. Details of the zero-order suppression procedure can be found in refs. [35,36].

It should be noted that, in practical digital holography experiments, the reconstructed image quality can also be influenced by several noise sources and image-degradation factors associated with coherent illumination, hologram recording, and numerical reconstruction. Camera-related noise includes shot noise, readout noise, dark noise, and quantization noise introduced by the finite bit depth of the image sensor. In addition, the pixel fill factor and nonuniform detector response may affect the recorded hologram intensity distribution and thus slightly modify the reconstructed amplitude and phase. Since coherent laser illumination was used in the experiment, speckle interference is also an important noise component and may introduce local intensity fluctuations in the recorded hologram and reconstructed image. Moreover, depending on the holographic recording geometry and the separation of diffraction orders in the Fourier domain, residual zero-order components, conjugate-image terms, and twin-image artifacts may further degrade the reconstructed image quality. In this study, proper exposure adjustment, zero-order suppression, and Fourier domain filtering were used to reduce these influences during hologram reconstruction. However, a complete quantitative decomposition of these noise contributions is beyond the main scope of the present work. Therefore, the experimental results are used to validate the proposed space-variant imaging field analysis under the employed recording conditions, while the above noise-related factors should be considered as practical limitations in digital holographic image formation.

### 3.2. Plane Wave Illumination: Simulation and Experiment

**Figure 4:**
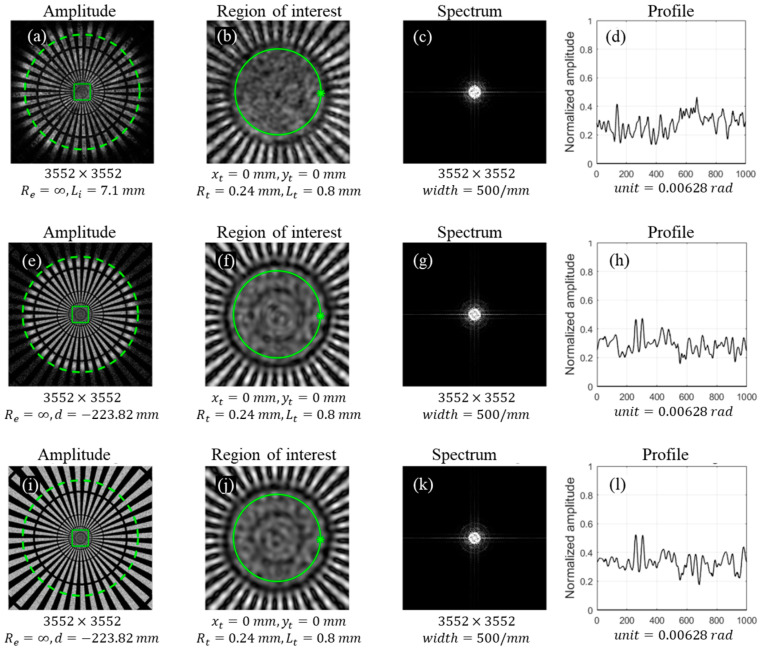
Experimental and theoretical results obtained when the center of the Siemens star coincides with the optical axis. (**a**–**d**) Experimental reconstruction; (**e**–**h**) theoretical simulation using Equation (3); and (**i**–**l**) theoretical simulation using Equation (1). Each row presents the amplitude image, region of interest, spectrum, and amplitude profile along the selected circular sampling path. The dashed circles in (**a**,**e**,**i**) indicate the projection boundary of the exit pupil, and the green squares indicate the regions of interest shown in (**b**,**f**,**j**), respectively. The profiles in (**d**,**h**,**l**) are the normalized sampling curves extracted along the sampling rings in (**b**,**f**,**j**), respectively, starting from the marked point on the right-hand side and proceeding counterclockwise for one full revolution.

Figure 4 presents the comparison of the experimental reconstruction and theoretical simulations for the case where the center coordinates of the Siemens star image in the image plane are $\left(x_{t}=0\;\mathrm{mm},y_{t}=0\;\mathrm{mm}\right)$. Figure 4a–d show the experimental results, Figure 4e–h show the theoretical simulation results calculated using Equation (3), and Figure 4i–l show the theoretical simulation results calculated using Equation (1). In each row, the panels present the amplitude image, the enlarged region of interest, the corresponding spectrum, and the normalized amplitude profile extracted along the selected circular sampling path, respectively. Figure 4a shows the amplitude of the digitally reconstructed holographic image, where the dashed circle indicates the projection boundary of the exit pupil. To more closely examine the relationship between resolution and spectral distribution, a small square region centered on the Siemens star image, with a side length of $L_{xy}=$ 400 pixels (0.8 mm), was cropped and enlarged, as shown in Figure 4b. A sampling ring with a radius of $R_{t}=$ 0.24 mm is also indicated in Figure 4b. To analyze the spectrum of the complex amplitude within this small square region, the complex amplitude outside the square in Figure 4a was set to zero, and its spectrum amplitude was then calculated using the Fourier transform. To more clearly visualize the spectral distribution, amplitude clipping was applied because the spectral amplitudes in the low-frequency region were relatively high. Specifically, all spectral amplitudes exceeding 1/100 of the central zero-order spectral amplitude were saturated and displayed in white. Figure 4d shows the normalized sampling curve extracted along the sampling ring in Figure 4b, starting from the marked point on the right-hand side and proceeding counterclockwise for one full revolution. The normalized amplitude profile was used to evaluate the resolvability of the Siemens star at the selected radial position. In this profile-based evaluation, adjacent bright and dark spokes were regarded as resolvable when their periodic modulation remained distinguishable along the circular sampling path. Therefore, the profile provides a quantitative one-dimensional representation for comparing the resolution characteristics of the experimental result and the theoretical simulations based on Equations (1) and (3).

Using the corresponding parameters, the theoretical simulation based on Equation (3) is shown in Figure 4e–h. In the simulation, a spherical wave with a wavefront radius of $R_{e}=$ 100,000 mm was used to approximate plane-wave illumination. To approximately reflect possible local phase perturbations under coherent illumination, a random phase perturbation ranging from 0 to π was introduced to the sampling points in the transparent regions of the Siemens star. Since the random phase used in the theoretical simulation cannot be expected to correspond exactly to the random phase variations occurring in the experiment, this treatment was used only as a simplified phenomenological model, rather than an exact reproduction of the experimental speckle pattern. Comparison of Figure 4a,e shows that the theoretical simulation obtained using Equation (3) agrees well with the experimentally reconstructed image. Theoretical analysis and numerical calculations further demonstrate that Equation (1) cannot correctly calculate the amplitude of the image field in this case. This is because the transfer function $P\left(-\lambda d_{pi}f_{x},-\lambda d_{pi}f_{y}\right)$ in Equation (3) can be interpreted as a spatial filter associated with diffraction of the ideal image over a finite propagation distance. Therefore, Equation (3) can accurately calculate the complex wavefront of the image field for an arbitrarily given exit pupil. It can also be seen that, owing to the spatial filtering effect, the reconstructed image decreases rapidly with increasing $r_{i}$ in the region near the projection boundary of the exit pupil. As a result, no resolvable Siemens star line patterns can be obtained along the selected sampling ring in either reconstruction image. In addition, the spectral distribution of the complex wavefront within the selected square region exhibits circular symmetry, indicating that the resolution is identical in all directions.

For comparison, Figure 4i–l show the theoretical simulation calculated using Equation (1). A comparison between Figure 4a,e,i shows that Equation (1) fails to accurately describe the amplitude distribution of the image field. This discrepancy arises because the amplitude transfer function $P\left(-\lambda d_{pi}f_{x},-\lambda d_{pi}f_{y}\right)$ in Equation (1) serves as a low-pass filter for the ideal image spectrum [29]. Consequently, the result calculated using Equation (1) produces a complete image extending over the entire reconstruction plane, which is inconsistent with the experimentally reconstructed image and the Equation (3)-based simulation. These comparisons demonstrate that Equation (3) provides a more appropriate description of the complex image-field wavefront for the present coherent imaging system.

**Figure 5:**
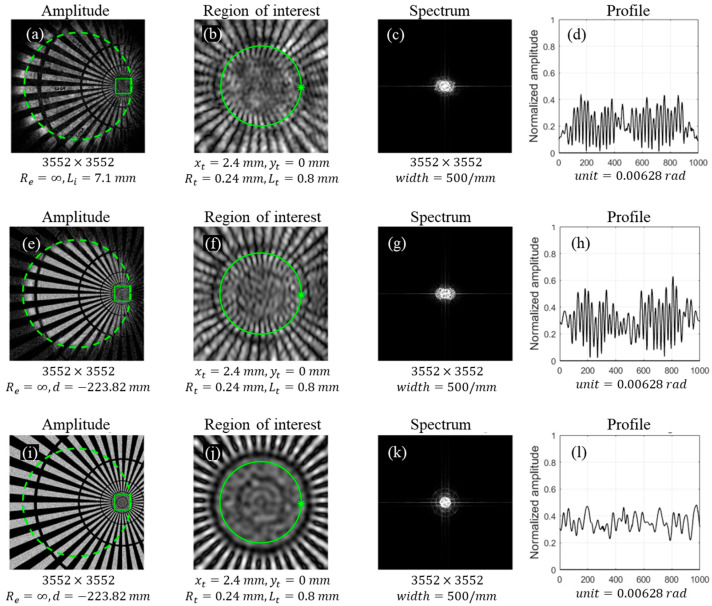
Comparison of the experimental reconstruction and theoretical simulations when the center coordinates of the Siemens star are (2.40 mm, 0 mm). (**a**–**d**) Experimental results; (**e**–**h**) theoretical simulation results obtained using Equation (3); and (**i**–**l**) theoretical simulation results obtained using Equation (1). Each row presents the amplitude image, enlarged region of interest, corresponding spectrum, and normalized amplitude profile along the selected circular sampling path. The dashed circles in (**a**,**e**,**i**) indicate the projection boundary of the exit pupil, and the green squares indicate the regions of interest shown in (**b**,**f**,**j**), respectively. The profiles in (**d**,**h**,**l**) are the normalized sampling curves extracted along the sampling rings in (**b**,**f**,**j**), respectively, starting from the marked point on the right-hand side and proceeding counterclockwise for one full revolution.

For Figure 4, the full field images shown in panels (a), (e), and (i) have a size of 3552 × 3552 pixels, corresponding to the full image sensor area used in the experiment and theoretical simulation. The enlarged regions shown in panels (b), (f), and (j) have a size of 400 × 400 pixels. The same pixel settings were used for the corresponding full field and enlarged region images in the related figures. Following the display format of figures, Figure 5 presents comparisons between the experimental and theoretical simulated images for the cases where the center coordinates of the Siemens star image in the image plane are (2.40 mm, 0 mm).

It can be observed from Figure 5 that the theoretically simulated image obtained using Equation (3) agrees well with the experimental image. In both the experimental results shown in Figure 5a–d and the Equation (3)-based simulation shown in Figure 5e–h, the spectrum in the observation region exhibits a horizontally broadened elliptical distribution, as shown in Figure 5c,g. This characteristic reflects the spectral distribution predicted by Equation (7). When the center of the Siemens star approaches the projection boundary of the exit pupil in the horizontal direction, corresponding to the case of θ= 0 in Equation (7), the spectrum reaches its maximum value $\frac{D_{pi}}{\lambda \left|d_{pi}\right|}$, which is twice that given by Equation (2). Under this condition, clear vertical line structures exceeding the Rayleigh resolution limit can be observed on the upper and lower sides of the sampling ring. In contrast, when θ=±π/2, Equation (7) reaches its minimum value, and unclear line structures can be observed along the selected sampling ring. For $\left|\theta \right|<\pi /2$, the spectral value varies continuously between these two extremes, and correspondingly, resolvable line structures gradually appear along the observation ring. These results not only provide strong experimental verification of Equation (7), but also further support the previous analysis regarding the limitations of Equation (3).

Figure 5i–l present the theoretically simulated image obtained based on Equation (1). Compared with the experimental results shown in Figure 5a–d, the Equation (1)-based simulation is clearly inconsistent with the experimental observation. This inconsistency can be attributed to the fact that the imaging calculation does not meet the applicability condition of Equation (1), which requires the object size to be smaller than one quarter of the incident pupil diameter [29]. Therefore, in the subsequent analysis, Equation (3) is used as the main theoretical model for comparison with the experimental results.

**Figure 6:**
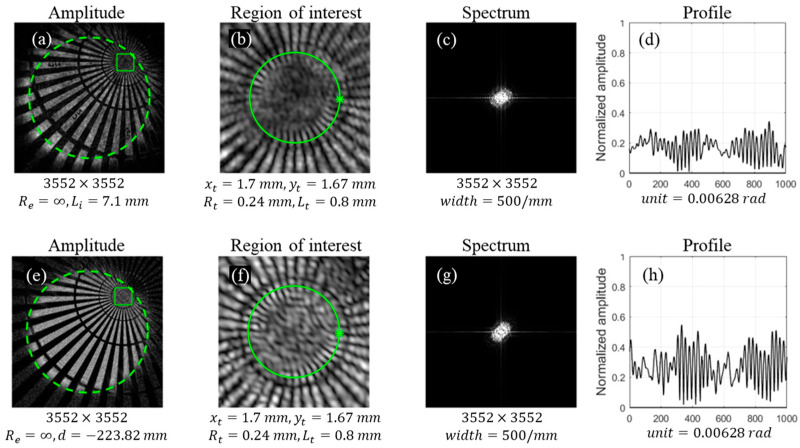
Comparison of the experimental reconstruction and theoretical simulations when the center coordinates of the Siemens star are (1.70 mm,1.67 mm). (**a**–**d**) Experimental results; (**e**–**h**) theoretical simulation results obtained using Equation (3). Each row presents the amplitude image, enlarged region of interest, corresponding spectrum, and normalized amplitude profile along the selected circular sampling path. The dashed circles in (**a**,**e**) indicate the projection boundary of the exit pupil, and the green squares indicate the regions of interest shown in (**b**,**f**), respectively. The profiles in (**d**,**h**) are the normalized sampling curves extracted along the sampling rings in (**b**,**f**), respectively, starting from the marked point on the right-hand side and proceeding counterclockwise for one full revolution.

To further verify the above analysis, Figure 6 presents the experimental measurements and theoretical simulations for the case where the center coordinates of the Siemens star image in the image plane are (1.70 mm, 1.67 mm). It is evident that, as the center of the Siemens star shifts toward the upper-right direction, the spectral distribution within the selected square region becomes broadened along the same direction, as shown in Figure 6c,g. In the observation region, the highest resolution is obtained in the direction perpendicular to the displacement, whereas the lowest resolution occurs in the direction parallel to the displacement. For the other directions, the resolution varies continuously between these two extremes. These results once again provide experimental verification of Equation (7).

### 3.3. Spherical Wave Illumination: Simulation and Experiment

**Figure 7:**
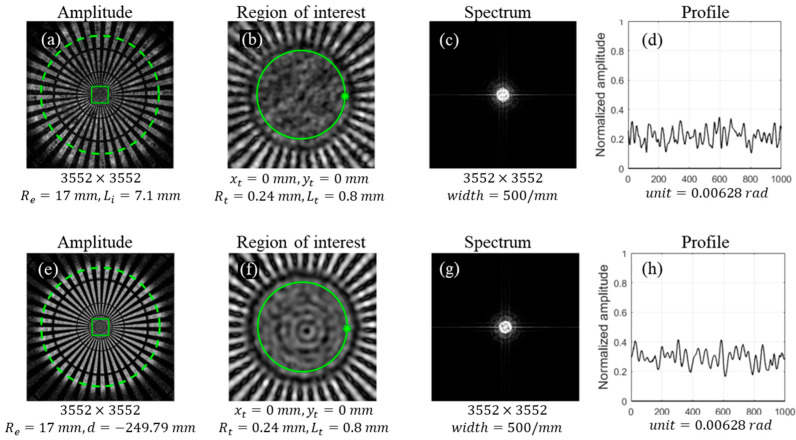
Comparison of the experimental and theoretical simulations obtained at $R_{e}=17\;mm$ when the center of the Siemens star coincides with the optical axis. (**a**–**d**) Experimental results; (**e**–**h**) theoretical simulation results obtained using Equation (3). Each row presents the amplitude image, enlarged region of interest, corresponding spectrum, and normalized amplitude profile along the selected circular sampling path. The dashed circles in (**a**,**e**) indicate the projection boundary of the exit pupil, and the green squares indicate the regions of interest shown in (**b**,**f**), respectively. The profiles in (**d**,**h**) are the normalized sampling curves extracted along the sampling rings in (**b**,**f**), respectively, starting from the marked point on the right-hand side and proceeding counterclockwise for one full revolution.

**Figure 8:**
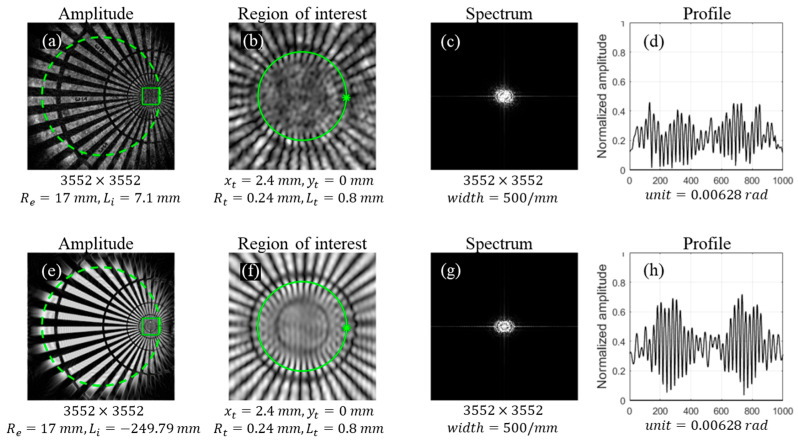
Comparison of the experimental and theoretical simulations obtained at $R_{e}=17\;mm$ when the center coordinates of the Siemens star are (2.40 mm, 0 mm). (**a**–**d**) Experimental results; (**e**–**h**) theoretical simulation results calculated using Equation (3). Each row presents the amplitude image, enlarged region of interest, corresponding spectrum, and normalized amplitude profile along the selected circular sampling path. The dashed circles in (**a**,**e**) indicate the projection boundary of the exit pupil, and the green squares indicate the regions of interest shown in (**b**,**f**), respectively. The profiles in (**d**,**h**) are the normalized sampling curves extracted along the sampling rings in (**b**,**f**), respectively, starting from the marked point on the right-hand side and proceeding counterclockwise for one full revolution.

**Figure 9:**
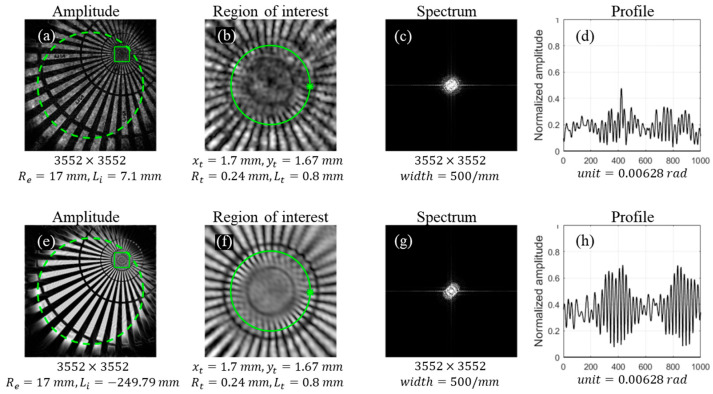
Comparison of the experimental and theoretical simulations obtained at $R_{e}=17\;mm$ when the center coordinates of the Siemens star are (1.70 mm, 1.67 mm). (**a**–**d**) Experimental results; (**e**–**h**) theoretical simulation results calculated using Equation (3). Each row presents the amplitude image, enlarged region of interest, corresponding spectrum, and normalized amplitude profile along the selected circular sampling path. The dashed circles in (**a**,**e**) indicate the projection boundary of the exit pupil, and the green squares indicate the regions of interest shown in (**b**,**f**), respectively. The profiles in (**d**,**h**) are the normalized sampling curves extracted along the sampling rings in (**b**,**f**), respectively, starting from the marked point on the right-hand side and proceeding counterclockwise for one full revolution.

Further experimental and theoretical simulation studies were conducted under spherical wave illumination with different wavefront radius. In the experimental setup, a lens $L_{0}$ was inserted, and its position was adjusted along the optical axis to realize a spherical wave illumination condition. Using the same presentation format as in the preceding figures, the case of an illumination wavefront radius of $R_{e}=17\;mm$ was investigated. Figure 7 shows the comparisons between the experimental results and the theoretical simulations when the center of the Siemens star coincides with the optical axis. Figure 8 presents the corresponding comparisons for the case where the center coordinates of the Siemens star in the image plane are (2.40 mm, 0 mm). Figure 9 shows the comparisons between the experimental and theoretical simulated images for the case where the center coordinates of the Siemens star in the image plane are (1.70 mm, 1.67 mm).

Comparison of the above figures shows that, under spherical wave illumination with $R_{e}=$ 17 mm, the theoretical simulations remain in good agreement with the experimental measurements. However, compared with the images obtained under plane wave illumination, it is easier to observe that the detectable image on the detection plane becomes significantly broadened. To facilitate a quantitative investigation of this effect, a comprehensive discussion will be given after the study on optimized spherical wave illumination is presented.

### 3.4. Optimized Spherical Wave Illumination: Simulation and Experiment

**Figure 10:**
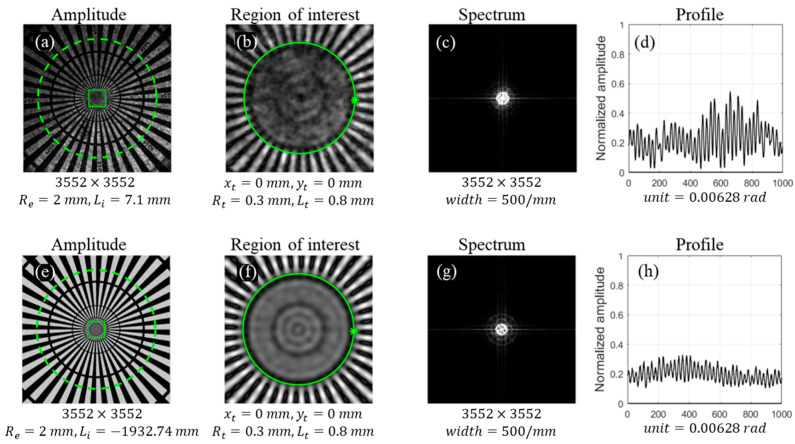
Comparison of the experimental and theoretical simulations obtained at $R_{e}=2\;mm$ when the center of the Siemens star coincides with the optical axis. (**a**–**d**) Experimental results; (**e**–**h**) theoretical simulation results obtained using Equation (3). Each row presents the amplitude image, enlarged region of interest, corresponding spectrum, and normalized amplitude profile along the selected circular sampling path. The dashed circles in (**a**,**e**) indicate the projection boundary of the exit pupil, and the green squares indicate the regions of interest shown in (**b**,**f**), respectively. The profiles in (**d**,**h**) are the normalized sampling curves extracted along the sampling rings in (**b**,**f**), respectively, starting from the marked point on the right-hand side and proceeding counterclockwise for one full revolution.

**Figure 11:**
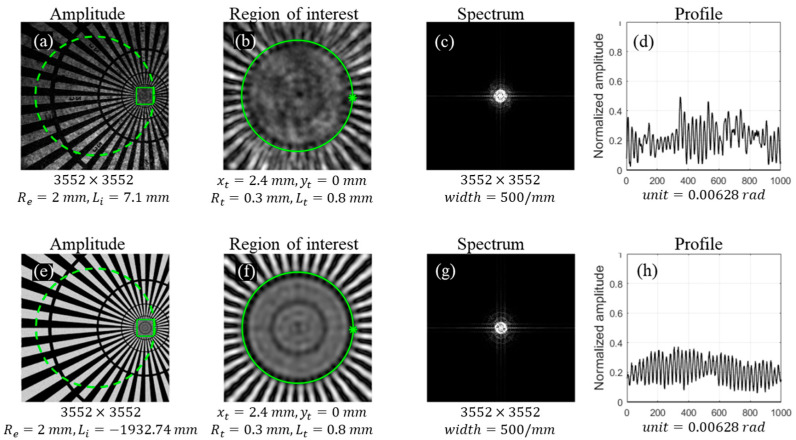
Comparison of the experimental and theoretical simulations obtained at $R_{e}=2\;mm$ when the center coordinates of the Siemens star are (2.40 mm,0 mm). (**a**–**d**) Experimental results; (**e**–**h**) theoretical simulation results calculated using Equation (3). Each row presents the amplitude image, enlarged region of interest, corresponding spectrum, and normalized amplitude profile along the selected circular sampling path. The dashed circles in (**a**,**e**) indicate the projection boundary of the exit pupil, and the green squares indicate the regions of interest shown in (**b**,**f**), respectively. The profiles in (**d**,**h**) are the normalized sampling curves extracted along the sampling rings in (**b**,**f**), respectively, starting from the marked point on the right-hand side and proceeding counterclockwise for one full revolution.

**Figure 12:**
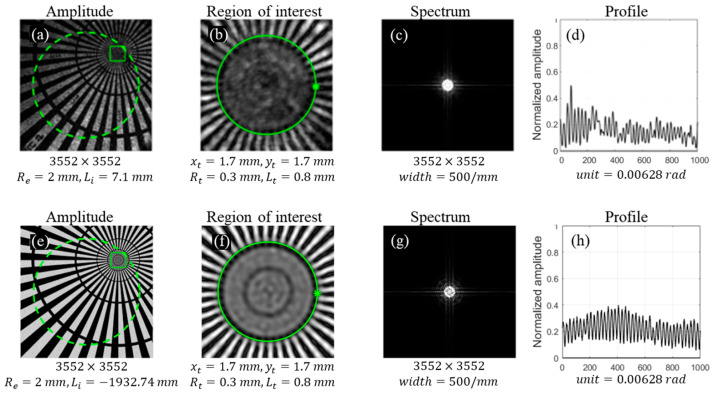
Comparison of the experimental and theoretical simulations obtained at $R_{e}=2\;mm$ when the center coordinates of the Siemens star are (1.70 mm,1.70 mm). (**a**–**d**) Experimental results; (**e**–**h**) theoretical simulation results calculated using Equation (3). Each row presents the amplitude image, enlarged region of interest, corresponding spectrum, and normalized amplitude profile along the selected circular sampling path. The dashed circles in (**a**,**e**) indicate the projection boundary of the exit pupil, and the green squares indicate the regions of interest shown in (**b**,**f**), respectively. The profiles in (**d**,**h**) are the normalized sampling curves extracted along the sampling rings in (**b**,**f**), respectively, starting from the marked point on the right-hand side and proceeding counterclockwise for one full revolution.

Using the same optical configuration, Equation (11) gives an optimized illumination wavefront radius of $R_{e}=$ 1.70 mm. In the experiment, the position of lens $L_{0}$ (focal length = 100 mm) was adjusted to achieve spherical wave illumination conditions close to the optimized value. Figure 10, Figure 11 and Figure 12 compare the experimental results with the theoretical simulations for the case where the illumination wavefront radius is approximately $R_{e}=2\;mm$.

When the illumination wavefront radius approximately satisfies Equation (11), $\left|d\right|$ in Equation (4) tends to infinity. Under this condition, the Fourier transform result approaches the spectrum of the ideal image, and the transfer function approximately behaves as a low-pass filter of the image spectrum. Consequently, the detector plane is able to capture the complete image, and the spectral distribution in the image plane becomes more uniform.

Furthermore, as $\left|d\right|\to \infty$, the second term on the right-hand sides of Equations (7) and (8) approaches zero. Therefore, the increase in resolution associated with the spectral enhancement that occurs when the observation region moves away from the optical axis is no longer evident. Instead, the overall resolution of the image plane is reduced.

The experimental results shown below are in good agreement with this theoretical analysis. When a sampling ring with a radius of 0.24 mm was used, no resolvable line features could be observed in the amplitude distribution at any position. To better examine the resolution variation in different observation regions, a sampling ring with a radius of 0.30 mm was adopted instead. Figure 10 shows the comparisons between the experimental results and the theoretical simulations when the center of the Siemens star coincides with the optical axis. Figure 11 presents the corresponding comparisons for the case where the center coordinates of the Siemens star in the image plane are (2.40 mm, 0 mm). Figure 12 shows the comparisons between the experimental and theoretical simulated images for the case where the center coordinates of the Siemens star in the image plane are (1.70 mm, 1.70 mm).

Figure 10, Figure 11 and Figure 12 show that, under spherical-wave illumination with $R_{e}=2\;mm$, the theoretical simulations agree well with the experimental measurements, and the detector is able to capture the complete image. To quantitatively analyze this phenomenon, substituting ${f=-R}_{e}=-2\;mm$, −17 mm, and −10,000 mm into $d={\left(\frac{C-1/\left(Af\right)}{A}-\frac{1}{d_{pi}}\right)}^{-1}$ gives d= −1419.25 mm, −249.79 mm, −223.8 mm respectively. Clearly, the magnitude $\left|d\right|$ increases as the illumination wavefront radius increases. When $R_{e}=$ 2 mm, the second term on the right-hand side of Equation (7) is very small, so the resolution shows no significant variation across different observation regions in the image plane. In addition, the Fourier transform in Equation (4) can be approximated as the spectrum of the ideal image. Therefore, according to Equation (3), the transfer function $P\left(-\lambda d_{pi}f_{x},-\lambda d_{pi}f_{y}\right)$ can effectively transmit the low-frequency components associated with the object morphology, allowing the detection plane to record a more complete image.

## 4. Discussion

The theoretical and experimental results presented above indicate that inserting the aperture stop AP into the optical system reduces the size of the exit pupil and leads to a smaller value of the parameter d in the spectral expression. Consequently, the image-plane spectrum becomes more sensitive to the observation position, which allows the derived spectral expression of the image field to be verified more intuitively.

In fact, the spectral expression of the image field is also valid for imaging systems without an aperture stop. In the absence of the aperture stop, the effective aperture stop is located at the objective lens plane, with a diameter of $2NAf_{0}=8\;mm$. After being imaged by lens, this aperture stop forms an exit pupil with a diameter of 90 mm at a position 2205 mm behind the lens. Therefore, the distance from the exit pupil to the image plane is given by $d_{pi}=d_{s}-2205\;mm=-2033.6\;mm.$ Under this condition, $d_{min}=1.22\lambda d_{pi}/D_{pi}\approx 0.02\;mm$ and $r_{min}=40d_{min}/\pi =0.22\;mm$. To better observe the variation in resolution with the observation position, the amplitude variation along a circular ring with a sampling radius of $R_{t}=0.75r_{min}\approx 0.17\;mm$ on the image plane was investigated.

**Figure 13:**
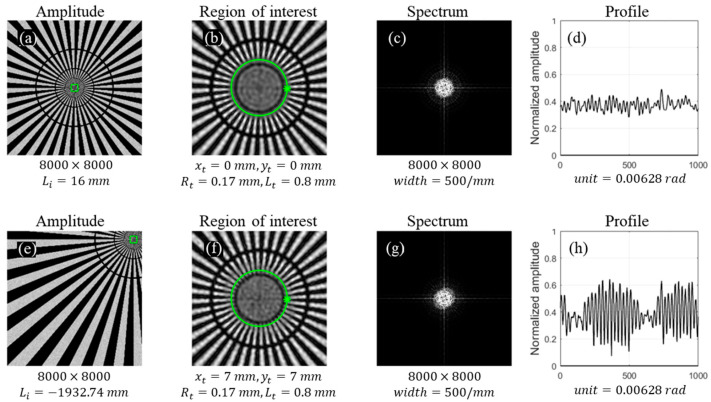
Theoretical simulation results obtained using Equation (3) under plane-wave illumination for different Siemens star positions. (**a**–**d**) Simulation results obtained when the center of the Siemens star coincides with the optical axis; (**e**–**h**) simulation results obtained when the center coordinates of the Siemens star are (7 mm, 7 mm). Each row presents the amplitude image, enlarged region of interest, corresponding spectrum, and normalized amplitude profile along the selected circular sampling path. The green squares in (**a**,**e**) indicate the regions of interest shown in (**b**,**f**), respectively. The profiles in (**d**,**h**) are the normalized sampling curves extracted along the sampling rings in (**b**,**f**), respectively, starting from the marked point on the right-hand side and proceeding counterclockwise for one full revolution.

The detector was set as an 8000 × 8000 pixel array. A spherical wave with $R_{e}=100,000\;mm$ was used to approximate plane-wave illumination on the object plane. Following the same image representation used in this study, Figure 13 shows the theoretically simulated images calculated using Equation (3) when the center coordinates of the Siemens star on the image plane are (0 mm, 0 mm) and (7 mm, 7 mm), respectively.

For the investigated case, the numerical calculation gives *d* = 2033.31 mm. This value indicates that the variation in the image-plane spectrum, or equivalently the resolution, with respect to the observation position is smaller than that in the case with the aperture stop. Nevertheless, a comparison with Figure 13 still shows that the resolution of the image field away from the center of the image plane is higher than that near the optical axis. This trend is also supported by the contrast values extracted from the normalized amplitude profiles in Figure 13d,h. The contrast was evaluated from the normalized amplitude profile along the selected circular sampling path. When the center of the Siemens star coincides with the optical axis, as shown in Figure 13d, the profile contrast is approximately 0.10. In contrast, when the center coordinates of the Siemens star are (7 mm, 7 mm), as shown in Figure 13h, the profile contrast increases to approximately 0.45. This increase indicates that the Siemens star line patterns become more distinguishable for the off-axis observation region. Since the detector window is located within the projected region of the exit pupil, the variation in the spectrum and resolution across different observation regions on the image plane still follows Equation (7).

**Figure 14:**
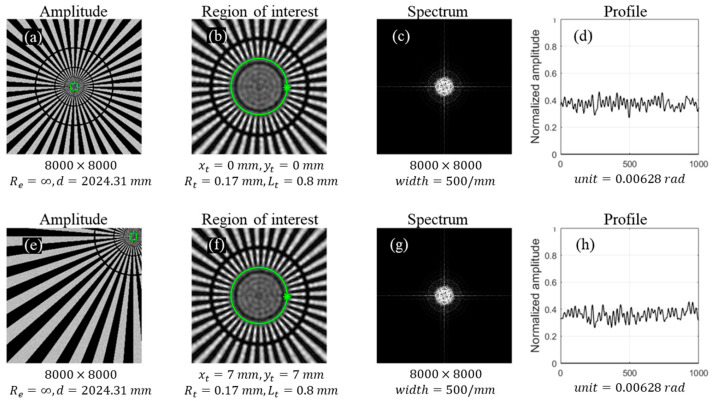
Theoretical simulation results obtained using Equation (1) under plane-wave illumination for different Siemens star positions. (**a**–**d**) Simulation results obtained when the center of the Siemens star coincides with the optical axis; (**e**–**h**) simulation results obtained when the center coordinates of the Siemens star are (7 mm, 7 mm). Each row presents the amplitude image, enlarged region of interest, corresponding spectrum, and normalized amplitude profile along the selected circular sampling path. The green squares in (**a**,**e**) indicate the regions of interest shown in (**b**,**f**), respectively. The profiles in (**d**,**h**) are the normalized sampling curves extracted along the sampling rings in (**b**,**f**), respectively, starting from the marked point on the right-hand side and proceeding counterclockwise for one full revolution.

The formula for calculating the image field amplitude distribution in Equation (1), which was derived in ref. [29] using an approximate treatment of the impulse response, has been widely recognized as a classical theoretical model. To compare it with the numerical simulations shown in Figure 13, the corresponding calculated images obtained using Equation (1) are presented in Figure 14, respectively.

It is evident that the image resolution obtained using Equation (1) is independent of both the observation position and the observation direction, as indicated by the nearly consistent contrast values without an obvious increase. This result is consistent with the physical implication of the spectral distribution formula, Equation (2), which was derived from Equation (1) in ref. [29]. According to Equation (2), the image-plane spectrum and the corresponding resolution remain unchanged with respect to the observation position and direction. However, this prediction does not agree with experimental observations in coherent optical imaging, where the resolution is closely related to the spatial position and illumination direction.

In contrast, the complex-amplitude calculation formula for coherent imaging introduced in this study, namely Equation (3), has been extensively verified by experiments [31,32,33]. With the continued development of image sensor technology, coherent imaging and inspection techniques based on large-area photodetector arrays are expected to become increasingly important. However, when a large-area photodetector is used, direct calculation based on Equation (3) may become difficult on computers with limited memory capacity. To address this issue, the image field can be divided into several smaller square regions and expressed as a stitched image field composed of these subregions.

For a small square region centered at $\left(-A\xi_{0},-A\eta_{0}\right)$, with a side length of N pixels and a physical width of $\left|AL\right|$, the method described in Appendix A can be adopted. By defining the optical field on the object plane as $rect\left(\frac{\xi -\xi_{0}}{L},\frac{\eta -\eta_{0}}{L}\right)U_{0}(\xi ,\eta )$, the corresponding calculation formula can be derived as

(14) $$U\left(x+A\xi_{0},y+{A\eta }_{0}\right)=exp\left\{\frac{jk}{2d_{pi}}\left[{\left(x+A\xi_{0}\right)}^{2}+{\left(y+{A\eta }_{0}\right)}^{2}\right]\right\}\times \;F^{-1}\left\{F\left\{-\frac{1}{A}rect\left(\frac{x}{AL},\frac{y}{AL}\right)U_{0}\left(\frac{x+A\xi_{0}}{AL},\frac{y+{A\eta }_{0}}{AL}\right)exp\left[j\frac{k}{2}\left(\frac{C}{A}-\frac{1}{d_{pi}}\right)\left({\left(x+A\xi_{0}\right)}^{2}+{\left(y+{A\eta }_{0}\right)}^{2}\right)\right]\right\}\times P\left(-\lambda d_{pi}f_{x},-\lambda d_{pi}f_{y}\right)\right\}$$

According to this expression, the Fourier transform and inverse Fourier transform can be computed with a sampling size of only N × N. Therefore, for coherent imaging with a large-area photodetector array, the image field in any specified region of the image plane can be calculated using a computer with limited memory capacity. For example, for the case shown in Figure 13, the ideal image can be divided into four quadrants, each with a sampling size of 4000 × 4000. The complete image field with an overall sampling size of 8000 × 8000 can then be obtained through four separate calculations.

From the perspective of imaging system design, these results provide useful guidance for the analysis and optimization of coherent imaging systems. For applications requiring more uniform resolution across the image field, the curvature radius of the illumination wavefront should be selected close to the optimized condition. Conversely, for applications requiring enhanced local resolution, it may be advantageous to place the observation region away from the center of the image plane and utilize the direction-dependent spectral enhancement effect. These findings indicate that both the illumination condition and the detection position should be carefully designed to obtain high-quality measurement results.

In addition to the theoretical and computational considerations discussed above, various approaches have been investigated to further improve the quality of digital holographic inspection [20,21]. The present results provide a theoretical basis for selecting the illumination object wave and the detection position according to the characteristics of the inspected object, thereby supporting improved coherent imaging and measurement performance. The generalizability of the proposed method can also be considered from this perspective. Since the formulation relates the spectral distribution at the image plane to the system parameters, observation position, and illumination wavefront, it is not limited to the specific DHM setup used in this experiment. In principle, the method can be extended to other coherent or holographic imaging systems by substituting the corresponding wavelength, exit pupil size, propagation distance, imaging geometry, and illumination conditions. Nevertheless, when applying the method to a different system configuration, the relevant optical parameters should be recalculated and the predicted space-variant spectral behavior should be validated under the corresponding experimental conditions.

In practical digital holographic reconstruction, the image quality may also be affected by several noise sources and reconstruction artifacts. Coherent illumination can introduce speckle noise, which produces local intensity fluctuations in the recorded hologram and reconstructed amplitude image [37]. In addition, conjugate-image artifacts and zero-order terms may overlap with the desired image information if the off-axis separation or filtering condition is not sufficient [38]. Digital sensor noise, including shot noise, fixed-pattern noise, readout noise, and quantization noise, may further influence the recorded hologram intensity and reconstructed image quality [39]. Signal saturation should also be avoided because saturated pixels distort the interference fringe modulation and may introduce reconstruction errors [40]. In the present experiment, proper exposure adjustment, zero-order suppression, and Fourier domain filtering were used to improve the hologram recording and reconstruction quality. Nevertheless, the present work focuses on the theoretical analysis and experimental validation of the space-variant spectral distribution and resolution behavior of coherent imaging systems. Therefore, a complete quantitative noise model is not included here. These noise-related factors should be considered in future applications when applying the proposed method to practical digital holographic inspection systems.

The application boundaries of the proposed method should also be noted. Since the derivation is based on a 2 × 2 optical ray-transfer matrix, the Collins formula, and the Fresnel diffraction integral, the present theoretical results are mainly applicable to rotationally symmetric coherent imaging systems satisfying the paraxial approximation. For systems with strong nonparaxial propagation, severe aberrations, non-rotational symmetry, strong scattering, or vectorial polarization effects, further extension and validation of the model would be required.

## 5. Conclusions

This work presents a comprehensive theoretical and experimental investigation of the spectral properties of the image field in coherent imaging systems. A general formulation capable of accurately calculating the complex wavefront at the image plane is developed, and explicit expressions describing spectral variation with respect to illumination conditions, observation position, and direction are derived. Experimental validation using digital holographic microscopy demonstrates excellent agreement with theoretical predictions. The results show that coherent imaging systems cannot be adequately described by conventional linear space-invariant models. Instead, both spectral distribution and spatial resolution exhibit strong dependence on position, direction, and illumination wavefront curvature. In addition, a practically applicable computational expression is derived to improve the feasibility of complex wavefront calculations for image fields sampled over large arrays. From an application perspective, the proposed analysis provides useful guidance for the design and optimization of coherent imaging and digital holographic systems, particularly when resolution uniformity across a large field of view is required. By quantitatively describing the spatially variant spectral distribution in the image plane, the proposed framework can help evaluate the influence of illumination wavefront curvature, exit-pupil configuration, and target position on local resolution. Therefore, it may be useful for large field digital holographic microscopy, microstructure and wafer inspection, biological cell observation, and other precision optical metrology applications in which local resolution consistency and reliable field-dependent image interpretation are important. Future works will further extend this framework to practical samples and application-specific measurement tasks, including the evaluation of measurement accuracy, field of view consistency, and sample-dependent imaging performance. These findings provide an important theoretical foundation for advanced coherent imaging system design and computational imaging applications.

## Figures and Tables

**Figure A1:**
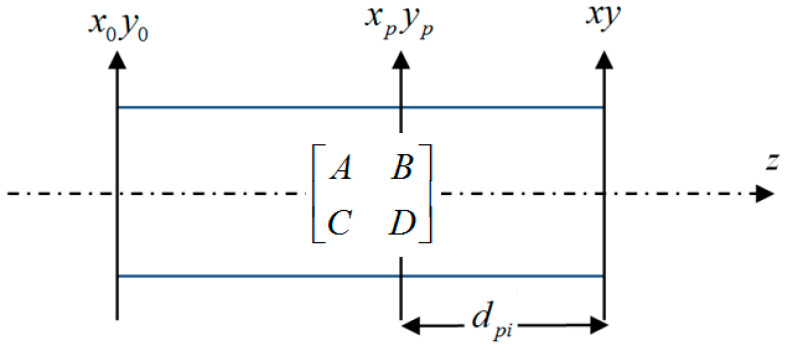
Diffraction-limited imaging system.

## Data Availability

The dataset underlying the results presented in this paper is not publicly available at this time but may be obtained from the authors upon reasonable request.

## Appendix A. Imaging Calculations of a Coherent Imaging System

### Appendix A.1. Impulse Response of Coherent Imaging System

Figure A1 is a schematic diagram of an imaging system composed of multiple optical elements. The system is assumed to be described by a 2 × 2 optical matrix $\left[\begin{array}{cc} A & B\\ C & D \end{array}\right]$. A Cartesian coordinate system *O-xyz* is defined, with the *z*-axis aligned with the optical axis of the system. The object plane, the exit pupil plane, and the image plane are denoted as $x_{0}y_{0},x_{p}y_{p},$ and xy, respectively. The distance from the exit pupil plane to the image plane is denoted as $d_{pi}$.

The imaging field resulting from a point source $\delta (x_{0}-\xi ,y_{0}-\eta )$ on the object plane can be expressed using the Collins formula as follows:

(A1) $$u_{\delta }\left(\xi ,\eta ;x,y\right)=\frac{exp\left(jkL\right)}{j\lambda B}\times \iint_{-\infty }^{\infty }\delta (x_{0}-\xi ,y_{0}-\eta )\times exp\left\{\frac{jk}{2B}\times \left[A\left(x_{0}^{2}+y_{0}^{2}\right)+D\left(x^{2}+y^{2}\right)-2\left(x_{0}x+y_{0}y\right)\right]\right\}{dx}_{0}{dy}_{0}$$

where $j=\sqrt{-1}$, k=2π/λ, λ is working wavelength, and L represents the optical path length along optical axis.

For an imaging system, the optical matrix from the object plane to the image plane satisfies the following condition:

(A2) $$\left[\begin{array}{cc} A & B\\ C & D \end{array}\right]=\left[\begin{array}{cc} A & 0\\ C & 1/A \end{array}\right]$$

Since the matrix element B=0, to address the issue of a zero denominator in Equation (A1), let ${Ax}_{0}=x_{a}$ and ${Ay}_{0}=y_{a}$, and noting that AD−BC=1, Equation (A1) can be rewritten as:

(A3) $$u_{\delta }\left(\xi ,\eta ;x,y\right)=exp\left(jkL\right)exp\left[j\frac{kC}{2A}\left(x^{2}+y^{2}\right)\right]\times \frac{1}{j\lambda BA}\iint_{-\infty }^{\infty }\frac{1}{A}\delta (\frac{x_{a}}{A}-\xi ,\frac{y_{a}}{A}\;-\eta )exp\left\{j\frac{k}{2BA}\left[{\left(x_{a}-x\right)}^{2}+{\left(y_{a}-y\right)}^{2}\right]\right\}{dx}_{a}{dy}_{a}$$

The condition B=0 can be regarded as the limiting case where B→0. By comparing the above expression with the well-known Fresnel diffraction integral, it is evident that as B→0, the expression represents an ideal image magnified by a factor of A, undergoing diffraction over an infinitesimally small distance BA, and multiplied by a quadratic phase factor. Thus, we have:

(A4) $$u_{\delta }\left(\xi ,\eta ;x,y\right)=\underset{\mathrm{BA}\to 0}{\lim}u_{\delta }\left(\xi ,\eta ;x,y\right)=exp\left(jkL\right)exp\left[j\frac{kC}{2A}\left(x^{2}+y^{2}\right)\right]\frac{1}{A}\delta \left(\frac{x}{A}-\xi ,\frac{y}{A}-\eta \right)$$

This result indicates that, in the absence of diffraction limitations, the image that reaches the image plane remains an ideal image point represented by a delta function δ.

Since $u_{\delta }\left(\xi ,\eta ;x,y\right)$ can also be regarded as the diffraction field on the image plane formed by the exit pupil under non-diffraction-limited conditions and propagated over a distance $d_{pi}$, the optical field $U_{\delta }\left(\xi ,\eta ;x_{p},y_{p}\right)$ on the exit pupil plane that does not pass through the exit pupil can be obtained by applying the inverse Fresnel diffraction operation:

(A5) $$U_{\delta }\left(\xi ,\eta ;x_{p},y_{p}\right)=-\frac{exp\left(-jkd_{pi}\right)}{j\lambda d_{pi}}\iint_{-\infty }^{\infty }u_{\delta }\left(\xi ,\eta ;x,y\right)exp\left\{-j\frac{k}{2d_{pi}}\left[{\left(x-x_{p}\right)}^{2}+{\left(y-y_{p}\right)}^{2}\right]\right\}dxdy$$

By substituting the relevant quantities into Equation (A5), and using the “filtering” property of the delta function, while ignoring the constant phase factor, we obtain:

(A6) $$U_{\delta }\left(\xi ,\eta ;x_{p},y_{p}\right)=-\frac{A}{\lambda d_{pi}}exp\left[j\frac{kC}{2}A\left(\xi^{2}+\eta^{2}\right)\right]exp\left\{-j\frac{k}{2d_{pi}}\left[{\left(A\xi -x_{p}\right)}^{2}+{\left(A\eta -y_{p}\right)}^{2}\right]\right\}$$

Let the exit pupil function be $P\left(x_{p},y_{p}\right)$. The optical field reaching the image plane through the exit pupil can be expressed using the Fresnel diffraction integral as:

(A7) $$h_{c}\left(\xi ,\eta ;x,y\right)=\frac{exp\left(jkd_{pi}\right)}{j\lambda d_{pi}}\iint_{-\infty }^{\infty }U_{\delta }\left(\xi ,\eta ;x_{p},y_{p}\right)P\left(x_{p},y_{p}\right)\times exp\left\{j\frac{k}{2d_{pi}}\left[{\left(x_{p}-x\right)}^{2}+{\left(y_{p}-y\right)}^{2}\right]\right\}{dx}_{p}{dy}_{p}$$

Substituting Equation (A6) into the above expression, and letting $f_{x}=\frac{x_{p}}{\lambda d_{pi}},f_{y}=\frac{y_{p}}{\lambda d_{pi}}$ we obtain:

(A8) $$h_{c}\left(\xi ,\eta ;x,y\right)=-Aexp\left[j\frac{k}{2}\left(\frac{C}{A}-\frac{1}{d_{pi}}\right)\left(A^{2}\xi^{2}+A^{2}\eta^{2}\right)\right]exp\left[j\frac{k}{2d_{pi}}\left(x^{2}+y^{2}\right)\right]{\;\times \;h}_{p}\left(x-A\xi ,y-A\eta \right)$$

In the above expression,

(A9) $$h_{p}\left(x,y\right)=\iint_{-\infty }^{\infty }P\left(\lambda d_{pi}f_{x},\lambda d_{pi}f_{y}\right)exp\left[-j2\pi \left(xf_{x}+yf_{y}\right)\right]{df}_{x}{df}_{y}$$

Up to this point, the impulse response of the coherent imaging system has been derived.

### Appendix A.2. Imaging Field in a Diffraction-Limited Imaging System

Let the optical field on the object plane be $U_{0}\left(\xi ,\eta \right)$. According to linear system theory, the optical field on the image plane can be expressed as a superposition integral using the impulse response of the imaging system:

(A10) $$U\left(x,y\right)=\iint_{-\infty }^{\infty }U_{0}\left(\xi ,\eta \right)h_{c}\left(\xi ,\eta ;x,y\right)d\xi d\eta$$

Substituting Equation (A8) into the above expression yields:

(A11) $$U\left(x,y\right)=-Aexp\left[j\frac{k}{2d_{pi}}\left(x^{2}+y^{2}\right)\right]\times \iint_{-\infty }^{\infty }U_{0}\left(\xi ,\eta \right)exp\left[j\frac{k}{2}\left(\frac{C}{A}-\frac{1}{d_{pi}}\right)\left(A^{2}\xi^{2}+A^{2}\eta^{2}\right)\right]\times h_{p}\left(x-A\xi ,y-A\eta \right)d\xi d\eta$$

Let $x_{a}=A\xi ,y_{a}=A\eta$, then

(A12) $$U\left(x,y\right)=exp\left[j\frac{k}{2d_{pi}}\left(x^{2}+y^{2}\right)\right]\times \iint_{-\infty }^{\infty }-\frac{1}{A}U_{0}\left(\frac{x_{a}}{A},\frac{y_{a}}{A}\right)exp\left[j\frac{k}{2}\left(\frac{C}{A}-\frac{1}{d_{pi}}\right)\left(x_{a}^{2}+y_{a}^{2}\right)\right]\times h_{p}\left(x-x_{a},y-y_{a}\right)d\xi d\eta$$

Multiplying both sides of Equation (A12) by $exp\left[-\frac{jk}{2d_{pi}}\left(x^{2}+y^{2}\right)\right]$ and then taking the Fourier transform of both sides yields:

(A13) $$F\left\{U\left(x,y\right)exp\left[-\frac{jk}{2d_{pi}}\left(x^{2}+y^{2}\right)\right]\right\}=F\left\{-\frac{1}{A}U_{0}\left(\frac{x}{A},\frac{y}{A}\right)exp\left[j\frac{k}{2}\left(\frac{C}{A}-\frac{1}{d_{pi}}\right)\left(x^{2}+y^{2}\right)\right]\right\}F\left\{h_{p}\left(x,y\right)\right\}$$

Since $h_{p}\left(x,y\right)$ is the Fourier transform of the exit pupil function, we have:

(A14) $$F\left\{h_{p}\left(x,y\right)\right\}=P\left(-\lambda d_{pi}f_{x},-\lambda d_{pi}f_{y}\right)$$

Substituting Equation (A14) into Equation (A13) and applying the two-dimensional inverse Fourier transform to both sides of the equation, the expression of the optical field on the image plane can be readily obtained in terms of Fourier transform and its inverse.

(A15) $$U\left(x,y\right)=exp\left[\frac{jk}{2d_{pi}}\left(x^{2}+y^{2}\right)\right]\times F^{-1}\left\{F\left\{-\frac{1}{A}U_{0}\left(\frac{x}{A},\frac{y}{A}\right)exp\left[j\frac{k}{2}\left(\frac{C}{A}-\frac{1}{d_{pi}}\right)\left(x^{2}+y^{2}\right)\right]\right\}\times P\left(-\lambda d_{pi}f_{x},-\lambda d_{pi}f_{y}\right)\right\}$$
